# Supplementary material for: Ketoanalogues Supplemental Low Protein Diet Safely Decreases Short-Term Risk of Dialysis among CKD Stage 4 Patients
Source: Nutrients. 2022 Sep 28;14(19):4020. doi: 10.3390/nu14194020 (PMC9571353; doi:10.3390/nu14194020)
Supplement: Supplementary file 1 [file nutrients-14-04020-s001.zip › nutrients-1907001-supplementary.pdf]

Table S1. Baseline characteristics of patients receiving ketoanalogues supplemental low protein diet after IPTW and EM imputation.

**Variables**	Continuation	Discontinuation	STD	*P* value
Age, years	67.8 ± 13.9	67.9 ± 14.2	-0.01	0.88
Age ≥65 years	60.6%	61.2%	-0.01	0.85
Male	62.4%	63.1%	-0.01	0.80
Body mass index, kg/m^2	25.4 ± 17.0	24.9 ± 4.8	0.04	0.38
eGFR at index, ml/min/1.73m^2	20.9 ± 4.8	20.8 ± 4.5	0.01	0.87
Comorbidities				
Coronary artery disease	29.2%	26.9%	0.05	0.42
Hypertension	81.6%	82.3%	-0.02	0.80
Diabetes mellitus	52.5%	53.1%	-0.01	0.86
Atrial fibrillation	5.9%	6.7%	-0.03	0.62
Liver cirrhosis	8.5%	8.5%	<0.01	0.99
Peripheral artery disease	9.2%	9.3%	<0.01	1.00
Dementia	5.8%	6.0%	-0.01	0.90
Systemic lupus erythematosus	1.4%	1.2%	0.01	1.00
Hepatitis B infection	5.4%	5.5%	<0.01	0.90
Hepatitis C infection	4.0%	4.0%	<0.01	1.00
Heart failure hospitalization	7.8%	7.7%	<0.01	0.99
Myocardial infarction	7.3%	7.0%	0.01	0.91
Stroke	10.6%	11.0%	-0.01	0.84
No. of outpatient visits on nephrology in the previous year				0.95
0	13.9%	14.6%	-0.02	
1-5	58.8%	59.7%	-0.02	
6-10	21.4%	20.0%	0.03	
>10	5.9%	5.7%	0.01	
No. of outpatient visits on all departments in the previous year	12.8 ± 9.6	12.7 ± 8.9	0.01	0.82
Admission in the previous year	35.2%	37.3%	-0.04	0.49
Follow-up years	1.5 [0.7, 3.5]	1.3 [0.6, 2.9]	0.08	0.16

Abbreviation: IPTW, inverse probability of treatment weighting; EM, expectation-maximization; STD, standardized difference; eGFR, estimated glomerular filtration rate.

Data were presented as frequency (percentage), mean ± standard deviation or median [25th, 75th percentile].

Table S2. Medication and laboratory data at baseline of patients receiving ketoanalogues supplemental low protein diet after IPTW and EM imputation.

**Variables**	Continuation	Discontinuation	STD	*P* value
Medication at baseline				
ACEi/ARB	59.0%	59.9%	-0.02	0.76
Beta-blockers	26.3%	26.7%	-0.01	0.89
Calcium-channel blocker	45.2%	45.4%	<0.01	0.95
Mineralocortocoid receptor antagonis	8.2%	9.2%	-0.04	0.59
Loop diuretics	33.7%	33.8%	<0.01	0.92
Nitrates	12.8%	12.8%	<0.01	1.00
Vasodilator	6.7%	6.5%	0.01	0.90
Thiazide	8.4%	8.5%	<0.01	0.91
Antiplatelet agents	31.3%	32.6%	-0.03	0.65
NSAIDs	10.8%	10.8%	<0.01	1.00
Steroid	13.4%	13.4%	<0.01	1.00
Proton pump inhibitor	17.2%	17.4%	-0.01	0.88
Insulin	13.9%	14.6%	-0.02	0.74
Oral hypoglycemic agents	36.7%	37.4%	-0.01	0.85
Pentoxyfillin	44.0%	43.9%	<0.01	1.00
Sodium bicarbonate	10.4%	10.2%	0.01	0.92
Fibrate	5.4%	5.1%	0.01	0.89
Statin	39.6%	41.5%	-0.04	0.50
Laboratory data at baseline				
Blood urine nitrogen, mg/dL	43.2 ± 16.8	43.5 ± 18.8	-0.02	
Creatinine, mg/dL	2.9 ± 0.8	2.9 ± 0.8	<0.01	
Proteinuria group, mg/dL				0.92
Negative (0-4)	16.1%	15.2%	0.02	
Trace (5-29)	6.8%	6.4%	0.01	
≥1+ (≥30)	77.1%	78.3%	-0.03	
CO_2	23.0 ± 3.3	23.0 ± 3.7	<0.01	0.89
Potassium, mg/dL	4.4 ± 0.6	4.4 ± 0.7	-0.01	0.67
Sodium, mg/dL	138.8 ± 3.5	138.8 ± 3.5	<0.01	0.76
Calcium, mg/dL	8.9 ± 0.6	8.9 ± 0.6	<0.01	0.94
Phosphorus, mg/dL	3.9 ± 0.7	3.9 ± 0.7	-0.03	0.18
HDL, mg/dL	45.3 ± 10.3	45.0 ± 9.6	0.03	0.68
LDL, mg/dL	76.6 ± 45.7	76.2 ± 35.7	0.01	0.96
Total cholesterol, mg/dL	176.7 ± 33.6	175.4 ± 36.5	0.04	0.43
HbA1C, %	6.6 ± 1.3	6.6 ± 1.4	-0.02	0.92
Albumin, mg/dL	3.9 ± 0.5	3.9 ± 0.5	<0.01	0.79
Hemoglobin, g/dL	10.5 ± 1.7	10.5 ± 1.8	-0.01	0.99
Serum uric acid, mg/dL	7.2 ± 1.8	7.3 ± 1.8	-0.01	0.70

Abbreviation: IPTW, inverse probability of treatment weighting; EM, expectation-maximization; STD, standardized difference; ACEi/ARB, angiotensin-converting enzyme inhibitors/angiotensin receptor blocker; NSAIDs, non-steroidal anti-inflammatory drugs; LDL, low-density lipoprotein; HDL, high-density lipoprotein; HbA1C, glycated hemoglobin.

Data were presented as frequency (percentage), mean ± standard deviation or median [25th, 75th percentile].
